# Drs. Jackson and Morton—Discovery of Etherization

**Published:** 1850-08

**Authors:** 


*Drs. Jackson and Morton — Discovery of Etherization.*—It is stated that the Academy of Sciences at Paris have awarded $500 to Dr. C. T. Jackson, of Boston, for his observations and experiments on the anæsthetic effects produced by the inhalation of ether, and to Dr. Morton the same sum for having "introduced it into practice."

